# Vigorous vs. moderate exercise to improve glucose metabolism in inactive women with polycystic ovary syndrome and insulin resistance: a pilot randomized controlled trial of two home-based exercise routines

**DOI:** 10.1016/j.xfre.2023.12.004

**Published:** 2023-12-22

**Authors:** Ange Wang, Martha Noel, Jacob P. Christ, Jamie Corley, Nikolaus Lenhart, Marcelle I. Cedars, Heather Huddleston

**Affiliations:** a Division of Reproductive Endocrinology and Infertility, University of California San Francisco, San Francisco, California; b Reproductive Medicine Associates Northern California, San Francisco, California

**Keywords:** Exercise, PCOS, glucose, insulin, randomized controlled trial

## Abstract

**Objective:**

To study the impact of vigorous vs. moderate exercise on metabolic parameters in polycystic ovary syndrome (PCOS).

**Design:**

Randomized controlled trial.

**Setting:**

Unsupervised home-based exercise program.

**Patient(s):**

Patients with PCOS on the basis of the Rotterdam criteria with insulin resistance.

**Intervention(s):**

Participants were block randomized to a home-based exercise program of 75 minutes of vigorous exercise or 150 minutes of moderate exercise per week, for 8 weeks total.

**Main Outcome Measure(s):**

Changes in glucose, insulin, and insulin resistance.

**Result(s):**

In total, 36 participants were randomized, of whom 20 completed the study. The percentage changes from baseline at 4 and 8 weeks for fasting glucose, insulin, and homeostatic model assessment for insulin resistance did not significantly differ between the groups, except for the change in the 8-week glucose level, which was more favorable in the moderate arm (8.06% [standard deviation, 6.44%] in the vigorous group compared with −0.32% [standard deviation, 4.91%] in the moderate group). The absolute values of the main outcomes (fasting glucose, insulin, and homeostatic model assessment for insulin resistance) at baseline and 4 and 8 weeks did not significantly differ between trial arms. When assessing the change from baseline at 4 and 8 weeks, overall and within each trial arm, only the 8-week fasting glucose level was significantly greater than the baseline value in the vigorous arm (93.5 [95% confidence interval, 88.7–98.3] vs. 86.8 [95% confidence interval, 81.1–92.4]).

**Conclusion(s):**

Unsupervised short-term exercise programs may not achieve significant metabolic improvements in patients with PCOS, regardless of vigorous vs. moderate intensity. Future studies should investigate this question in larger sample sizes and longer or structured exercise programs.

**Clinical Trial Registration Number:**

ClinicalTrials.gov identifier, NCT02303470.

Polycystic ovary syndrome (PCOS), a common endocrine disorder with a prevalence of 7%–12%, is primarily characterized by hyperandrogenism, oligomenorrhea, and polycystic ovaries (1, 2). Although not required for diagnosis, individuals with PCOS also show increased rates of insulin resistance and other associated metabolic comorbidities (3). Insulin resistance appears to contribute to the underlying pathophysiology of PCOS, and indeed, many of the sequelae of PCOS can be improved by interventions that reduce insulin levels (4). For example, previous research has demonstrated that insulin-sensitizing medications, such as metformin, can improve ovulation patterns, hyperandrogenemia, and metabolic health (5, 6). Studies of lifestyle modifications, such as calorie reduction and/or increased physical activity, have also shown promise in improving metabolic parameters and, in some cases, restoring ovulatory function (7, 8, 9). As such, the current PCOS guidelines state that all patients with PCOS receive counseling on healthy lifestyle behaviors and that lifestyle interventions that incorporate both physical activity and diet strategies be recommended to those with excess weight to optimize health outcomes (10). With regard to specific recommendations for physical activity, the PCOS guidelines, in alignment with the current Department of Health and Human Services (DHHS) recommendations for all Americans, suggest a minimum of either 150 minutes of moderate activity or 75 minutes of vigorous activity or a combination of both (11, 12). However, the question naturally arises, from both providers and their patients, whether one of these options (moderate or vigorous activity) may achieve greater improvements in important outcomes.

High-intensity interval training (HIIT) is a form of vigorous exercise that combines short intervals of intense exercise with lower-intensity recovery periods (13). Although initially introduced as a training modality for high-performance athletes, more recent investigations have studied the therapeutic potential of HIIT in adults with cardiovascular disease, diabetes, obesity, and metabolic syndrome (14, 15, 16, 17, 18, 19). This growing body of work suggests that when compared with moderate exercise, HIIT shows greater improvement in aerobic capacity, maximal oxygen consumption, indices of insulin resistance, hyperglycemia, and lipid profiles. High-intensity interval training has also been compared with moderate-intensity exercise in patients with type 2 diabetes mellitus and has shown a reduction in hyperglycemia, although these data have been controversial in terms of clinical significance (20, 21). A recent small (n = 29) randomized controlled trial (RCT) of overweight women with PCOS completed in Australia also found that HIIT resulted in greater improvements in insulin sensitivity than moderate-intensity continuous training (22). These results have yet to be confirmed in a general population of patients with PCOS in the United States.

Accordingly, we completed a pilot study to evaluate the relative benefits of two exercise programs for patients with PCOS: a short-duration, vigorous exercise program and a longer-duration, moderate exercise program, both requiring equivalent expenditure of metabolic units per week. We designed programs that could be completed in or around the home, requiring only 15–30 minutes per day, that would meet the minimum weekly requirements for physical activity, either 75 minutes of vigorous activity or 150 minutes of moderate activity. Our study design required patients to complete their assigned exercise plan on their own, without intensive support or monitoring. In this way, our study was designed to produce generalizable results about accessible and practical exercise strategies.

## Materials and methods

### Trial Oversight

This study was an RCT to evaluate the metabolic effects of implementing a structured but unsupervised exercise program in women with PCOS and insulin resistance, conducted at a single academic medical center. The protocol was approved by the University of California San Francisco Institutional Review Board, and the trial was registered on ClinicalTrials.gov before participant enrollment. Written informed consent was obtained from all participants before the start of the trial.

### Participants, Screening, and Recruitment

The study population consisted of currently inactive women with PCOS and insulin resistance. Participants were recruited from 2015 to 2022 through the University of California San Francisco specialty PCOS clinic, a multispecialty clinic staffed by a reproductive endocrinologist, dermatologist, psychologist, and dietitian with a goal of diagnosing and counsel patients about PCOS. The inclusion criteria were the following: diagnosis of PCOS as defined by the 2003 Rotterdam criteria (add Rotterdam citation); presence of insulin resistance as defined by homeostatic model assessment for insulin resistance (HOMA-IR) level of >2.0 or fasting insulin level of ≥12 mU/L; and current self-reported physical activity less than the DHHS recommendations before enrollment (either 75 minutes of vigorous activity, 150 minutes of moderate activity per week, or a combination of the 2) (11, 12). To make this determination, we used the International Physical Activity Questionnaire that all patients completed before their first clinical visit. The International Physical Activity Questionnaire collects self-reported physical activity information and allows for a quantification of moderate and vigorous activity minutes per week (23).

The exclusion criteria included the following: age of <18 or >50 years; body mass index (BMI) of >40 kg/m^2^; current tobacco use; pre-existing diagnosis of type 2 diabetes mellitus, uncontrolled hypertension (>140/90 mm Hg), or cardiovascular disease; presence of musculoskeletal injury or disease that would interfere with the patient’s ability to complete exercise program; current pregnancy or planning to attempt to conceive in the next 3 months; and physician judgment that the patient would be unable to complete exercise program. Participants who met the criteria were informed about the study, and those who chose to participate gave written informed consent.

As a pilot RCT, the primary goal was to identify any outcome differences between the two treatment arms. A total of 67 participants consented to the trial, 36 were randomized, and nine in the vigorous and 11 in the moderate groups completed the trial. Assuming a standard deviation (SD) in the change in fasting insulin level of 3 pmol/L (24) and at an alpha of 0.05, we would have 80% power to detect a difference in the change in the insulin level of 4 pmol/L on the basis of the number of participants who completed the trial.

### Randomization and Treatment Arm

Computer-generated block randomization to a home-based exercise program comprising either 75 minutes of vigorous exercise or 150 minutes of moderate exercise per week for 8 weeks total was completed. The block randomization was five blocks of four assignment slots each (two moderate and two vigorous), with the slots within each block in a randomized order of assigned presentation to make it as unpredictable and nonrepeating as possible to ensure that there is no pattern. Allocation concealment was completed; the clinical research coordinator was blinded to the order until the point of reviewing the protocol with the participant, at which point unblinding was necessary. All patients also received counseling about healthy diet strategies from a nutritionist according to standard clinic protocols. Participants in the high-intensity group were instructed in HIIT-specific exercises performed in 15 minutes per day, 5 days per week. Participants in the moderate-intensity group were instructed in brisk walking for 30 minutes per day, 5 days per week. To provide guidance regarding how to achieve activity of either a vigorous or moderate intensity, participants met in-person with an exercise physiologist to determine the target heart rates for the assigned exercise program. In particular, using age and resting heart rate, an exercise physiologist determined a target heart rate range for high-intensity or moderate-intensity exercise, using a formula modified from that of Karvonen et al. (25). Participants were instructed to make their best effort to adhere to their assigned exercise plan and target heart rate and received a wearable heart rate and activity tracker to for self-monitoring. Beyond the initial exercise physiologist meeting, no further outside supervision of the exercise program was performed.

### Measures

All participants underwent a standardized baseline evaluation before randomization. Patients were instructed to discontinue hormonally active medications for at least 1 month prior and metformin 2 weeks before laboratory and clinic evaluations. A standardized assessment of endocrine and fasting metabolic parameters including a 75-g oral glucose tolerance test was completed, and all participants through Quest laboratory examinations also underwent standardized anthropometric assessments, assessment for hirsutism, and pelvic ultrasound by a trained physician according to previously described protocols (26). Fasting glucose and insulin were measured at the completion of the 8-week intervention through Quest laboratory examinations.

### Analyses

The primary outcomes of the trial were the percentage change in fasting glucose, insulin, and HOMA-IR at 4 and 8 weeks, compared with the baseline values. HOMA-IR was calculated as fasting glucose × fasting insulin/405. Participants who dropped out before randomization or while completing the exercise program were not included in the final analysis because of the lack of follow-up laboratory data. Participants who completed the trial were analyzed per their initial treatment allocation arm, regardless of adherence to the program. The Student t-tests and Wilcoxon signed-rank tests were used to compare normally and non–normally distributed continuous variables respectively. As part of an exploratory analysis, univariate logistic regression was used to assess associations between our primary outcomes and age, BMI, Beck Depression Inventory scores, cholesterol, and systolic/diastolic blood pressure. All tests were 2-sided with significance at the alpha = 0.05 level. Data analysis was performed in STATA Version 16 (StataCorp, College Station, TX) and IBM SPSS Statistics Version 28.

## Results

**Figure 1:**
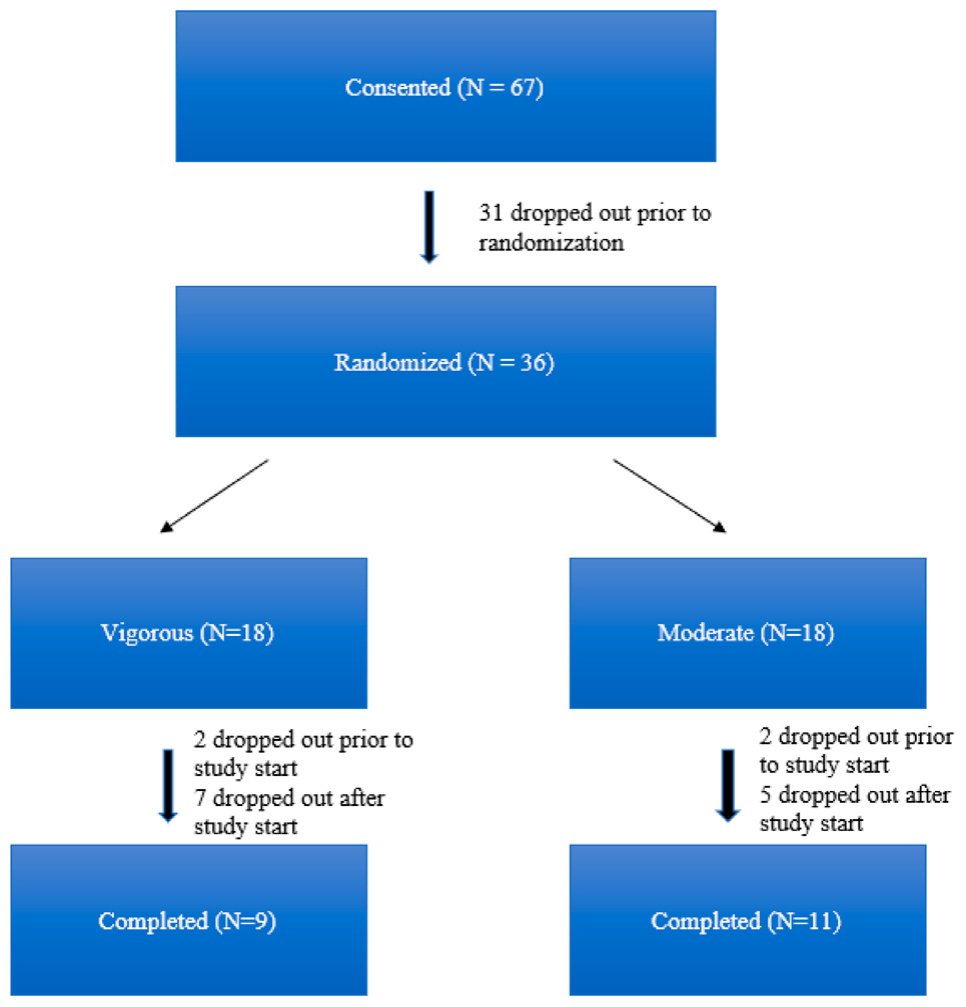
Participant information diagram.

A total of 67 patients consented to the trial, of whom 31 dropped out after consenting, leaving 36 participants who were initially randomized (Fig. 1). Of these participants, 19 completed the 8-week program, and one participant completed the first 4 weeks of the program and obtained 4-week laboratory examinations (moderate arm); these 20 participants were included in the final analysis. Of the 16 participants who dropped out after randomization, nine had been randomized to the vigorous group, and seven had been randomized to the moderate group. The reasons given for dropping out (Supplemental Fig. 1, available online) included time commitment (n = 8), desire to pursue a different exercise routine (n = 4), and injury sustained before beginning program (n = 1). Those who dropped out after randomization did not significantly differ from those who completed the study (Supplemental Table 1, available online).

In total, 11 patients completed the moderate exercise arm (including one who completed up to week 4), and nine completed the vigorous exercise arm. The baseline characteristics did not differ across groups (Table 1). The mean BMIs were in the obese range for both groups: 32.6 (SD, 7.5) kg/m^2^ for the moderate group and 33.7 (SD, 6.3) kg/m^2^ for the vigorous group (*P* = .74). Other metabolic metrics, including total cholesterol, high-density lipoprotein, triglycerides, and 2-hour insulin and glucose, were statistically similar between the groups at baseline.

**Table 1:** Baseline characteristics of patients with polycystic ovary syndrome randomized to moderate and vigorous exercise interventions.

Patient characteristics	Moderate (n = 11) Mean ± SD or n (%)	Vigorous (n = 9) Mean ± SD or n (%)	*P* value^a^
Age (y)	30.1 ± 6.3	32.4 ± 5.3	.40
BMI (kg/m^2^)	32.6 ± 7.5	33.7 ± 6.3	.74
Beck Depression Inventory score	5.9 ± 4.7	5.3 ± 4.6	.79
Total cholesterol (mg/dL)	189.9 ± 23.7	197.9 ± 31.7	.57
HDL (mg/dL)	59.3 ± 11.3	48.9 ± 9.3	.07
Triglycerides (mg/dL)	125.8 ± 60.1	128.7 ± 36.3	.91
2-h insulin (mIU/mL)	117.8 ± 88	151 ± 124.4	.56
2-h glucose (mg/dL)	113.2 ± 34.4	115.5 ± 23.5	.89
MFG score	13 ± 9	10 ± 6	.50
DHEAS (mg/dL)	372.6 ± 229.7	263.2 ± 134.3	.33
Androstenedione (ng/dL)	266.1 ± 108.2	124.5 ± 147.3	.08
Total testosterone (ng/dL)	50.4 ± 24.5	52.4 ± 25.1	.89
Free testosterone (pg/mL)	8.3 ± 7.2	5.2 ± 2.8	.38
SHBG (nmol/L)	28.7 ± 16.6	73.2 ± 103.9	.39
FNPO	22.9 ± 6.05	26.1 ± 13.8	.59
Ovarian volume (cc)	10.8 ± 5.3	6.8±4.7	.18
Oligoanovulation	6 (75.0)	4 (66.7)	.73
PCOM	7 (100)	4 (66.7)	.10
Clinical or biochemical HA	7 (87.5)	6 (100)	.37

*Notes:* BMI = body mass index; DHEAS = dehydroepiandrosterone sulfate; FNPO = follicle number per ovary; HA = hyperandrogenism; HDL = high-density lipoprotein; mFG = modified Ferriman-Gallwey; PCOM = polycystic ovarian morphology; SHBG = sex hormone binding globulin.

a *P* value derived from an independent sample t-test or the Mann-Whitney U test for continuous variables and the chi-square test for categorical variables.

Our primary outcomes of percentage change from baseline (by trial arm) are displayed in Table 2. The percentage change from baseline at 4 and 8 weeks for fasting glucose, insulin, and HOMA-IR did not significantly differ between the groups, with the exception of the change in the 8-week glucose level, which was more favorable in the moderate arm (8.06% [SD, 6.44%] in the vigorous group compared with −0.32% [SD, 4.91%] in the moderate group, *P* = .01).

**Table 2:** Percentage change from baseline for outcomes (4 and 8 weeks).

Outcome	Moderate		Vigorous		*P* value
	Mean percentage	SD	Mean percentage	SD	
4-wk glucose	−1.86%	11.00%	5.80%	6.99%	.10
8-wk glucose	−0.32%	4.91%	8.06%	6.44%	**.01**
4-wk insulin	1.98%	32.00%	14.32%	35.18%	.44
8-wk insulin	8.29%	40.80%	15.25%	32.96%	.70
4-wk HOMA-IR	1.76%	49.20%	21.10%	39.29%	.31
8-wk HOMA-IR	8.05%	40.74%	25.83%	41.73%	.38

*Note:* HOMA-IR = homeostatic model assessment for insulin resistance; SD = standard deviation.

When assessing change from baseline at 4 and 8 weeks, overall and within each trial arm, only the 8-week fasting glucose level was significantly greater than the baseline value in the vigorous arm (93.5 [95% CI, 88.7–98.3] compared with 86.8 [95% CI, 81.1–92.4]). All other values were not significantly different across the three timepoints (Table 3). The absolute values of the main outcomes (fasting glucose, insulin, and HOMA-IR) at baseline and 4 and 8 weeks did not significantly differ between the trial arms (*P* > .05 for all, Supplemental Table 2, available online).

**Table 3:** Baseline vs. 4-/8-week outcome comparisons (t-tests): mean (95% confidence interval).

	Overall			*P* values	
	Baseline	4 wk	8 wk	Baseline vs. 4 wk	Baseline vs. 8 wk
Glucose	89.5 (86.6–92.5)	90.5 (86.2–94.9)	92.3 (89.7–95.0)	.64	.063
Insulin	20.6 (15.4–25.8)	21.1 (15.1–27.0)	20.7 (15.5–25.8)	.81	.57
HOMA-IR	4.6 (3.4–5.7)	4.8 (3.2–6.3)	4.5 (3.2–5.7)	.69	.89

	Moderate arm			*P* values	
	Baseline	4 wk	8 wk	Baseline vs. 4 wk	Baseline vs. 8 wk
Glucose	91.5 (88.2–94.9)	89.7 (82.9–96.5)	91.4 (87.8–95.0)	.56	.79
Insulin	23.2 (16.0–30.5)	23.2 (13.6–32.9)	22.4 (15.0–29.7)	.99	.81
HOMA-IR	5.3 (3.6–7.0)	5.3 (2.7–8.0)	4.7 (2.7–6.6)	.99	.56

	Vigorous arm			*P* values	
	Baseline	4 wk	8 wk	Baseline vs. 4 wk	Baseline vs. 8 wk
Glucose	86.8 (81.1–92.4)	91.6 (85.2–98.1)	93.5 (88.7–98.3)	.06	**.007**
Insulin	17.0 (8.5–25.5)	18.1 (10.5–25.7)	18.5 (9.4–27.5)	.64	.42
HOMA-IR	3.6 (2.0–5.2)	4.0 (2.5–5.6)	4.2 (2.3–6.2)	.3	.19

*Note:* Mean (95% confidence intervals for all values). The exact baseline values vary slightly on the basis of metric; the values compared with those in 4 weeks are displayed. The nonparametric test (Wilcoxon sign-rank) similar results are not displayed. Bold *P*-values denote significance.

HOMA-IR = homeostatic model assessment for insulin resistance.

To investigate whether any of the baseline characteristics were predictive of change in metabolic factors, we performed a secondary analysis using the univariate analysis of percentage change from baseline for glucose/insulin/HOMA-IR (using linear regression) for all participants regardless of randomization. No significant correlation was noted with any of the predictors studied (age, BMI, systolic/diastolic blood pressure, total cholesterol, and Beck Depression Inventory score, *P* > .05 for all).

## Discussion

In an 8-week RCT comparing the effect of moderate vs. vigorous exercise for metabolic outcomes in patients with PCOS, we found that participants in the moderate exercise arm achieved a statistically significantly greater reduction in the fasting glucose levels, with no difference in the fasting insulin and HOMA-IR levels. Furthermore, the overall results for both arms were relatively modest, and we observed a high dropout rate, perhaps reflecting the challenges faced by patients who were not highly active at baseline in executing a home-based, unsupervised exercise routine.

The beneficial effects of exercise in the general population and in women with PCOS are well established (27). As such, lifestyle interventions have long been a mainstay of the recommendations made to patients with PCOS, including in the recent 2018 international guidelines (2). These guidelines, similar to the DHHS advice for all Americans, recommend a minimum of 75 minutes of vigorous or 150 minutes of moderate exercise per week (11). Within this framework, the two options are treated as equivalent. However, whether vigorous and moderate activities are truly equivalent or whether one may yield better results for patients with PCOS is unknown. A previous cross-sectional study of 326 patients with PCOS, of whom 56% met the DHHS guidelines for adequate physical activity, found that vigorous, but not moderate, activity was associated with reduced odds of metabolic syndrome, controlling for exercise volume (28). Similar results were obtained in a recent meta-analysis of 19 trials (including an RCT and non-RCTs of 777 women) on a variety of exercise interventions of at least moderate intensity (29). Although this meta-analysis did not include any head-to-head comparisons of moderate to vigorous, the investigators concluded that vigorous-intensity exercise may have the greatest impact on cardiorespiratory fitness, body composition, and insulin resistance but that a minimum of 120 minutes of vigorous intensity per week was needed to provide favorable health outcomes (29). Subsequent to the meta-analyses, Patten et al. (22) published the first RCT (24 completers) of vigorous-intensity (HIIT) vs. moderate-intensity exercise programs. This study entailed a supervised regimen, conducted thrice weekly with an exercise physiologist using an exercise bike ergometer, and found that vigorous exercise produced greater improvements in cardiorespiratory fitness, fasting glucose, and insulin sensitivity than moderate exercise (22).

To our knowledge, our study is now the second study (and the first in the United States) to directly compare moderate and vigorous exercise routines for patients with PCOS, with each arm requiring expenditure of equivalent metabolic units. Contrary to the trial by Patten et al. (22), we found slightly better outcomes, specifically change in the fasting glucose levels at 8 weeks, in the moderate exercise arm. In fact, our study reported that the glucose levels significantly increased in the vigorous group, with a small decrease in the moderate group. The reason for the improved results observed in the moderate group is not immediately clear, although it is possible that the vigorous exercise program in our trial may have generated a greater physical stress than moderate activity, leading to an increase cortisol level and subsequent dysglycemia (30). Alternatively, differential adherence to the exercise programs could be a factor. The study by Patten et al. (22) involved a highly supervised program, whereas our study entailed an unsupervised program that patients conducted at home after a single training session. It is possible that patients in the moderate exercise arm (which involved brisk walking) may have found the program less intimidating and more accessible, leading to better overall adherence and, therefore, greater exercise volume relative to the vigorous arm.

It is notable that most previous studies of exercise in PCOS involved structured exercise programs with direct oversight provided by study personnel (31, 32, 33, 34). This design addresses the narrow, scientific question of how exercise affects particular outcomes in PCOS; however, it can be difficult to generalize these results to what can be achieved in the “wild,” outside a supportive and well-resourced structure. In contrast, our study attempted to simulate the real-life scenario of a patient adopting a home-based exercise program as may be recommended by a healthcare provider during a typical clinical encounter. Our high dropout rate should be interpreted accordingly and, importantly, suggests that the common provider advice is not sufficient to precipitate meaningful changes in behavior. Furthermore, even for those who completed the program, improvements were modest at best. Although the benefits of exercise compared with no activity are well established for individuals with PCOS, our results do provide generalizable information that may temper both provider and patient expectations for meaningful short-term gains. Perhaps more importantly, from a public health perspective, our results suggest that the provision of (and payment for) supervised exercise therapy is required to achieve the benefits reflective of those achieved in most published reports. Additionally, the utilization of automated systems (e.g., artificial intelligence or tracking applications on mobile phones) may be useful in terms of filling the gap between supervised and unsupervised exercise programs.

### Strengths and Limitations

The strengths of the study include its RCT format, which is difficult to implement for exercise programs. Our study also used the exact DHHS guidelines for exercise in our study design because previous trials have used a variety of exercise programs and formats that are heterogeneous in nature and, therefore, difficult to generalize. There was also detailed information available on PCOS status because patients were seen in the multidisciplinary PCOS clinic and rigorously assessed with the Rotterdam criteria.

The limitations of this study include a high dropout rate among consented patients, which is likely at least partially due to the practical difficulties of adhering to an exercise program. Additional reasons for patient dropout are shown in Supplemental Figure 1, although most participants did not report a reason and never started study activities. Better understanding of reasons for not starting and/or dropout will improve ultimate success from a public health perspective. Those without any follow-up data were excluded from analyses. Given the exploratory nature of the study, high dropout rate, and small sample size, we believe that including these participants could add bias and make interpreting results more difficult. However, we acknowledge that uneven dropout may also have biased our results. In addition, we had limited compliance data because of unexpected technical issues with wearable tracking devices and lack of posttreatment survey data on exercise compliance. Therefore, we were unable to fully analyze adherence to the assigned treatment arm. We also did not have information on diet, which is known to contribute to metabolic outcomes and is an area for future investigation for patients with PCOS. Given that this was a pilot study, our sample size was small and not based on a prespecified sample size calculation. Furthermore, because of difficulty with recruiting for an exercise study and the coronavirus disease 2019 pandemic, recruitment took place over 7 years. Additional limitations, with regard to generalizability, include the inclusion criteria for insulin resistance and inactivity at baseline. Therefore, the results may not apply to patients who have normal metabolic indices or who were already performing some activity. Finally, there was not a control arm in the study design, and therefore, we can only draw conclusions about vigorous vs. moderate exercise but not overall exercise vs. no intervention.

## Conclusion

In conclusion, in an RCT of a short-term, unsupervised exercise routine, we found that both moderate and vigorous physical activity assignments yielded similar and relatively modest results. We note that adherence is a challenge to the investigation of exercise interventions, as evidenced by a high dropout rate. Our results, even for participants who completed the program, are far more modest than the results achieved in supervised research settings. This suggests that with only initial counseling/instruction and self-monitoring, it is difficult for patients to achieve measurable change in metabolic measures. These results could inform public health recommendations for physical activity and stimulate a discussion regarding how to effectively support long-term adherence to exercise recommendations in high-risk populations.

## Declaration of Interests

A.W. has nothing to disclose. M.N. has nothing to disclose. J.P.C. reports funding from Pacific Coast Reproductive Society outside the submitted work. J.C. has nothing to disclose. N.L. has nothing to disclose. M.I.C. reports funding from NIA; travel support and leadership role American Society of Reproductive Medicine - Past President, member of Executive Committee outside the submitted work. H.H. has nothing to disclose.
