# Supplementary material for: Vigorous vs. moderate exercise to improve glucose metabolism in inactive women with polycystic ovary syndrome and insulin resistance: a pilot randomized controlled trial of two home-based exercise routines
Source: F S Rep. 2023 Dec 22;5(1):80–6. doi: 10.1016/j.xfre.2023.12.004 (PMC10958708; doi:10.1016/j.xfre.2023.12.004)

| **Supplemental table 1: Participants that completed vs dropped out of the study.** | | |
| --- | --- | --- |
|  | Completed study (n=20) | Dropped out of study (n=16*) |
| Age (years) | 31.1±5.8 | 32±7.7 |
| BMI (kg/m^2^) | 32.5±7.6 | 33.7±7.2 |
| Total cholesterol (mg/dL) | 193.4±26.8 | 180.7±26.1 |
| HDL (mg/dL) | 54.811.5 | 47.510.2 |
| Triglycerides (mg/dL) | 127.1±49.5 | 136.3±61.2 |
| 2hour insulin (mIU/mL) | 128.9±98.3 | 133.7±126.5 |
| 2hour glucose (mg/dL) | 114.1±29.9 | 112.3±24.5 |
| MFG score | 12±8 | 13±7 |
| DHEAS (mg/dL) | 322.1±192.7 | 215±114.5 |
| Androstendione (ng/dL) | 207.1±140 | 153.1±69.7 |
| Total testosterone (ng/dL) | 51.2±23.7 | 46.4±26.1 |
| Free testosterone (pg/mL) | 7.1±6 | 7.4±5.7 |
| SHBG (nmol/L) | 45.8±65.3 | 32.8±27.9 |
| FNPO | 24.4±10 | 24.3±13.9 |
| Ovarian volume (cc) | 9±5.3 | 7.2±3 |
| Oligoanovulation | 10 (71) | 11 (79) |
| PCOM | 11 (85) | 7 (50) |
| Clinical or Biochemical HA | 13 (93) | 13 (93) |
| Phenotype |  |  |
| OA+PCOM | 1 (8) | 0 (0) |
| PCOM+HA | 3 (25) | 1 (9) |
| OA+HA | 1 (8) | 4 (36) |
| OA+HA+PCOM | 7 (58) | 6 (55) |
| *Data was not available for 2 participants that dropped out of the trial. Across all variables no significant differences (p<0.05) were seen between those that completed and dropped out of the study. BMI- body mass index, DHEAS- dehydroepiandrosterone sulfate, HDL- high density lipoprotein, mFG- modified Ferriman Gallwey, SHBG- sex hormone binding globulin, FNPO- follicle number per ovary, PCOM- polycystic ovarian morphology, HA- hyperandrogenism, OA- oligoanovulation | | |

| **Supplemental Table 2. Baseline, 4, 8 week values by trial arm** | |  |  | |  |  |
| --- | --- | --- | --- | --- | --- | --- |
|  |  |  |  | |  |  |
|  | Moderate Mean (SD) | Vigorous Mean (SD) | | p-value |  |  |
| Baseline insulin | 23.2 (10.8) | 16.4 (9.7) | | 0.16 |  |  |
| 4 week insulin | 23.2 (14.4) | 18.1 (9.1) | | 0.38 |  |  |
| 8 week insulin | 22.4 (10.3) | 18.5 (10.8) | | 0.45 |  |  |
| Baseline glucose | 91.5 (5) | 87.9 (7.2) | | 0.20 |  |  |
| 4 week glucose | 89.7 (10.1) | 91.6 (7.7) | | 0.66 |  |  |
| 8 week glucose | 91.4 (5.1) | 93.5 (5.8) | | 0.42 |  |  |
| Baseline HOMA-IR | 5.3 (2.6) | 3.5 (1.8) | | 0.10 |  |  |
| 4 week HOMA-IR | 6.3 (3.9) | 4 (1.9) | | 0.41 |  |  |
| 8 week HOMA-IR | 4.7 (2.9) | 4.2 (2.3) | | 0.73 |  |  |

**Supplemental Figure 1.**


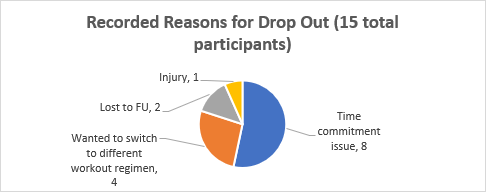

Supplement: Supplemental Materials [file mmc1.docx]
